# Robot-assisted surgery in space: pros and cons. A review from the surgeon’s point of view

**DOI:** 10.1038/s41526-021-00183-3

**Published:** 2021-12-21

**Authors:** Desirè Pantalone, Giulia Satu Faini, Francesca Cialdai, Elettra Sereni, Stefano Bacci, Daniele Bani, Marco Bernini, Carlo Pratesi, PierLuigi Stefàno, Lorenzo Orzalesi, Michele Balsamo, Valfredo Zolesi, Monica Monici

**Affiliations:** 1 Department of Experimental and Clinical Medicine, University of Florence (IT), Emergency SurgeryUnit- TraumaTeam, Emergency Dept–Careggi, University Hospital, Florence, Italy; 2 Department of Experimental and Clinical Medicine, University of Florence, Careggi University Hospital, Florence, Italy; 3 ASAcampus joint laboratory, ASA Research Division, Department of Experimental and Clinical Biomedical Sciences “Mario Serio”, University of Florence, Florence, Italy; 4 Department of Biology, Research Unit of Histology and Embriology, University of Florence, Florence, Italy; 5 Department of Experimental & Clinical Medicine, Section of Anatomy & Histology, Research Unit of Histology & Embryology - University of Florence, Florence, Italy; 6 Oncology Department, Breast Unit, Careggi University Hospital, Florence, Italy; 7 Department of Experimental and Clinical Medicine-University of Florence, Vascular Surgery Unit, Cardio -Thoracic and Vascular Dept-Careggi University Hospital, Florence, Italy; 8 Department of Experimental and Clinical Medicine-University of Florence, Cardiovascular Surgery Unit. Cardio-Thoracic and Vascular Dept -Careggi University Hospital, Florence, Italy; 9 Department of Experimental and Clinical Medicine, University of Florence, Florence, Italy; 10 Breast Unit, Oncology Department, Careggi University Hospital, Florence, Italy; 11 Kayser Italia srl, Livorno, Italy

**Keywords:** Three-dimensional imaging, Trauma

## Abstract

The target of human flight in space has changed from permanence on the International Space Station to missions beyond low earth orbit and the Lunar Gateway for deep space exploration and Missions to Mars. Several conditions affecting space missions had to be considered: for example the effect of weightlessness and radiations on the human body, behavioral health decrements or communication latency, and consumable resupply. Telemedicine and telerobotic applications, robot-assisted surgery with some hints on experimental surgical procedures carried out in previous missions, had to be considered as well. The need for greater crew autonomy in health issues is related to the increasing severity of medical and surgical interventions that could occur in these missions, and the presence of a highly trained surgeon on board would be recommended. A surgical robot could be a valuable aid but only inasfar as it is provided with multiple functions, including the capability to perform certain procedures autonomously. Space missions in deep space or on other planets present new challenges for crew health. Providing a multi-function surgical robot is the new frontier. Research in this field shall be paving the way for the development of new structured plans for human health in space, as well as providing new suggestions for clinical applications on Earth.

## Introduction

For more than 50 years space exploration has been growing, allowing new achievements in basic science and technology that proved useful also for Earth issues, in sectors like^1^ health care, space jobs creation, technological improvements to every day products, weather forecasts and communications, satellite data on climate change, and natural disaster prediction. For example, in 2020 Anderson et al.^2^ provided an overview on the new achievements of telerobotics, planetary science, and human space flight. In the communications sector, telepresence technology allows human exploration at multiple sites included those considered too dangerous for astronauts. On Earth, this technology shall support human exploration in hostile environments, lowering costs and risks.

Astronaut health, both during long-term space flights and/or settlements on another planet, is one of the topics under study. However, policies of a rapid return to Earth^3,4^, currently adopted on the International Space Station (ISS) in low Earth orbit (LEO) can no longer be the chosen option as the growing distances from Earth shall make impossible any kind of ground support. In this paper, we consider the role of robotic assisted surgery as a valuable help for astronauts in long-term missions and missions on another planet. An examination of the effects of weightlessness and absence of gravity, as well as signal delay and the level of crew autonomy complete the review.

In particular, it must be said that although the presence of a qualified experienced surgeon providing medical assistance and performing surgical procedures is desirable, it cannot be taken for granted^3,4^. The availability of a surgical robot could be useful, although the concept behind robot-assisted surgery is different from the one we are used to on Earth^5^. In fact the robotic surgical systems currently present in our Operative Room (OR) on Earth are too bulky and heavy for space flights and need room and assistance by qualified personnel to allow the operator to perform surgery.

These robots are master–slave teleoperated devices, but space research has been developing pre-programmed, more autonomous multi functional surgical robots, capable of performing procedures autonomously^5,6^. In addition to performing basic surgical procedures, such as suturing, they should provide diagnostic instrumentation and interpretation for ultrasound, Computed Tomography-scan (CT-scan) or Magnetic Resonance Imaging (MRI)^5,6^ and also be able to give support in anesthesia and vital-signs monitoring, as the Crew Medical Officer (CMO) could be alone in taking care of severely ill or injured astronauts.

## Framing topics

### Hints on microgravity (µg) and 0 gravity (0 g) effects on the physiology of the human body

Microgravity (μg) is the condition in which people or objects appear to be weightless^7^. Weightlessness is a condition where the accustomed physiological challenges due to the gravity vector, to which the human body is daily subject to on the Earth’s surface, are absent^7^. The normal 1 G condition affects the human body and the cardiovascular, pulmonary, neurovestibular and musculo skeletal systems present specific or particular sensitivity to it^3,7–10^. The human body shows several alterations due to microgravity effects^3,7–10^. There are no differences in physiological responses between microgravity in LEO and zero gravity beyond the planetary gravitational forces. Body fluids have a shift and the cardiovascular compensation produces are distribution of fluids with increased blood volume in the head and chest vessels. The reduction in heart work load during long-duration spaceflight due to the absence of gravity leads to a decrease in the overall myocardial mass^3,7,8^. Also the Musculo skeletal system is affected by the absence of conventional gravitational forces, showing atrophy of bones and supporting muscles, with predisposition to pathological fractures during physical activity or return to normal gravity^3,7^. Immune dysregulation is also present with leukocytosis during and after space flight, significant enough to produce increased susceptibility to bacterial and viral infections^3,7^. Moreover, adaptation to weightlessness produces a neurovestibular dysfunction known as space motion sikness^7^. Many other modifications occur during permanence in space^7^, due to different causes, like changes in physical activity, energy expenditure, limb modification following fluid shift and muscular changes. The anthropometry of the thorax and abdomen also changes: the chest presents a “barrel” shape with diaphragmatic elevation of one or two intercostal spaces, while the abdomen shows a “flattened” contour with rostral location of liver and spleen upon palpation^7^. Also the vertebral spine presents a change for the expansion of the intervertebral discs and the loss of the thoracolumbar curvature due to the unloading condition^7^.

**Fig. 1:**
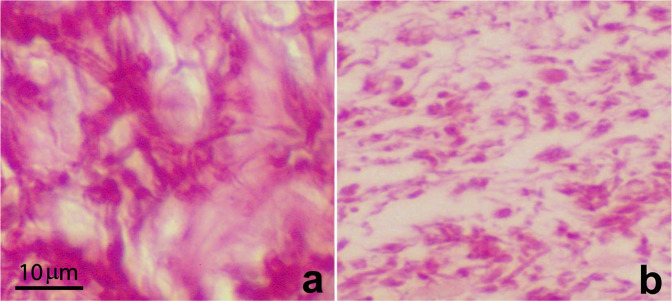
Effect of exposure to modeled microgravity in wound healing. The effect of exposure to modeled microgravity conditions in an in vivo model of wound healing (Hirudo Medicinalis): collagen fibers content at the wound site in 1 x g control (**a**) and microgravity-exposed sample (**b**). Exposure to modeled microgravity induces a significant decrease in collagen fibers content with evident disorganization of the fiber network. Picrosirius red staining.

Among other process is important to recall that wound healing is impaired in space^11,12^. Although the literature on wound healing in weightlessness is relatively poor, studies on animal models in immune cells, fibroblasts, endothelial and epithelial cells cultured both in real and in modeled µg conditions, show alterations in phagocytosis, adhesion/migration, apoptosis, proliferation, intercellular cross-talking, production of inflammatory mediators, extracellular matrix molecules, growth factors and so on^11,12^ (Fig. 1).

### Hints on surgical studies

While previously the probability of an inflight event that would require a surgical operation during long-duration mission was small, the advent of a lunar project, deep space exploration, and missions to another planet, i.e., Mars, has increased likelihood of traumatic events during space flights that may require surgery and, consequently, a change in space policy^12,13^. In fact urgent surgical diseases such as appendicitis and cholecystitis can occur without warning signs^3,14^ and virtually any unpredictable event that may occur outside the terrestrial orbit is a risk for the individual crew member, the whole crew and the mission^15^. In space, surgery requires dedicated protocols and devices for the maintenance of sterile surgical fields^16,17^, as well as adequate anesthesia and appropriate surgical techniques. This assumption is true both for open surgery, laparoscopic surgery and robot assisted minimal invasive surgery (RAMIS). However, laparoscopic and robotic surgery have the advantage of keeping the abdominal cavity closed, preventing the leakage of blood and fluids from inside it into the atmosphere of the spacecraft and contributing to the maintenance of body temperature^13^. Another concern besides the possible contamination of the spacecraft atmosphere is the need to protect the operative field from the particulate matter present in the spacecraft^3,4,13^. Maintenance of hemostasis, instrument deployment and containment of operator and patients are also necessary.

Other possibilities tested in space surgery were represented by the use of a closed surgical system (canopy)^18^ for surgery on anesthetized animals in parabolic flights to evaluate the behavior of arterial and venous bleeding, bleeding control and prevention of atmospheric contamination^14^. Studies on laminar flow devices to reduce the risk of wound contamination with pathogenic bacteria found on the skin and with colonies in the spacecraft atmosphere have been also conducted^3,6,18^. Regarding the containment of body fluids on the one hand and the prevention of contamination of the surgical field by environmental debris and bacteria on the other, laparoscopy and RAMIS are considered effective because they are able to separate the surgical field from the environment maintaining the integrity of the abdominal wall^4,19^. In all of these studies conducted on open surgery and laparoscopic surgery during parabolic flights and on the ISS^14,15,17^, surgical procedures were found to be feasible in space although more difficult to perform than on Earth^20,14^. Kirkpatrick et al.^19,13^ described the behavior of the abdominal wall in laparoscopy that is different at 0 g compared to what occurs at terrestrial gravity on an animal model (pig) during parabolic flights^19,13^. The abdominal wall of the pig showed a spontaneous pressure increase and a change in shape, from compressed to round ellipse, when entering weightlessness. In this study^19,13^ two different procedures for laparoscopic visualization were compared: one without gas insufflation associated to abdominal wall retraction through a dedicated device, and the other with standard15mmHG gas insufflation^19,13^. The conclusion was that gas insufflation improves visualization, and allows better intra-abdominal conditions for laparoscopic surgery^19,13^. For long duration exploration missions), Robertson et al. in 2020^21^ suggested a new approach to care. Considering that there is indeed an increased risk of traumatic injury in long-term missions, in planetary surface exploration, and in space walks, the Authors examined medical events with life saving potential, their potential impact on crew health and mission success and developed a list of non technical skills (NTSs) to be used in-flight medical management training^21^. Sudden cardiac arrest, smoke inhalation, toxic exposure, seizures, and penetrating eye injuries were the events with the greatest potential impact on missions, and NTS were found to be helpful for successful management. In addition these findings may have an application also on Earth for surgical and medical care in rural and remote locations^21^.

### Notes on the crew medical officer (CMO)

Currently, the CMO is just a crew member without medical background, with only 60 h of medical training^22^ as the risk of a severe injury or illness on the ISS^15^ is low. On the contrary, during long-term flights conditions such as appendicitis, peptic ulcer, intestinal obstruction are likely to occur even in healthy crew members^14^, in addition to traumatic injuries. The latter may impact on the mission to the extent that they may cause its failure^3,7,15,17^.

Since on long-term missions or missions to Mars, trauma and emergency surgical diseases are expected to require treatment on the spot, as returning to Earth would take ~9 months^4,15^ both the CMO and crew need to have more extensive surgical and medical capabilities^3,17,20,22,23^. A dedicated medical selection and training is, for now, the chosen policy for exploration class missions and on long-term space flights, the CMO should also have a trained medical assistant who may replace him/her if necessary^9,17,22–24^ (CMO severe injury, disease or death).

## Robotic surgery in space

Given that robotic surgery in space is the central topic of the review, some aspects need to be explored. One of them is communication issues. NASA Space Communications and Navigation (SCaN)^25^ program enables data exchange, with astronauts aboard the ISS, as well as with rovers on Mars, and the Artemis missions to the Moon. NASA has an extensive network of antennas over all continents to receive transmissions from space crafts and may also rely on relay satellites in order to get their data to the ground. The Tracking and Data Relay Satellites, will send data through orbiters around Mars, which forward the data to Earth. Bandwidth with various bands of electromagnetic frequencies are available with different capabilities.

Higher bandwidths can carry more data per second but a system to communicate with infrared lasers^26^ is expected to be launched in 2021 that shall revolutionize communications to and from space, topic that is not included in this review. For now, bandwidth is not the only constraint for data flow rates since communications don’t occur instantaneously: the speed of light is their limit, about 186,000 miles per second or 299,792 km per second. For spacecraft close to Earth, this time delay—or communications latency—is almost negligible. Farther from Earth, however, latency becomes a problem. When Mars is closer to Earth—about 35 million miles away, 56,327,000 Km—the delay is about 4 min. At their greatest distance—about 250 million miles away, 402,300,000 Km—the delay is around 24 min^25^. This means that communication varies from 4 to 24 min of delay to reach Mission Control. And another 4 to 24 min to receive its response depending upon the distance between the two planets^25^.

In addition, the quality of communications transmission travelling long distances or through atmosphere, can deteriorate, distorting the message. Also sun or other planets radiation can interfere^25^. The accumulation of these adverse conditions can seriously affect robotic surgery in space, compromising the signal from and to Earth and, consequently, any real time action^25^.

### Hints on telemedicine

Many studies have been done to test the possibility of telementoring in space surgery. Long-distance telementoring may be the only available form of medical aid in space exploration. Its applications depend on the different mission types and distances from Earth and on the quality of communication technology^25,27–30^. Though most technological advancements covered in this section are applicable to Low Earth (LEO)or Geostationary Earth (GEO) orbits, at longer distances such as on Moon or Mars, none of the current communication technologies can reduce latency^25^. In a publication of 2011 Haidegger et al.^31^, made a review on the various possibilities of teleassistance in relationship to the distance. Telemedicine can be on line and off line depending on the link quality, and offers various modalities: *store-and-forward telemedicine* is exclusively a one-way communication at a time, however improvable through *remote monitoring* or *interactive telepresence*^31^. In case the connection is not reliable, a remote surgeon can still communicate with a local colleague thanks to the video and voice feed from the operating room. This modality, or telementoring, is also called “*consulting telemedicine*” or “*consultancy telehealth*”^31^.

### NASA extreme environment mission operations, the “NEEMO missions”

Telementoring experiments have been conducted in the NEEMO missions^32^, a corner stone program whereby NASA has sent astronauts to live in Aquarius under water laboratory since 2001^32^. The 7th mission took place in 2004^32,33^, the 9th mission in 2006^32,34^ and the 12th in 2007^32,35^, with focus on telemedicine. Each usually lasted 7–14 days. Aquarius is the only world’s undersea research station, with a habitat approximately the size of ISS, large enough to accommodate six members (aquanauts). A dedicated buoy supplies the module with power, life support and communications via umbilicals^32^.

NEEMO missions were expressly designed for testing telemedicine assisted by surgical robots. For example, the AESOP robot (ZEUS) was used in the 7th mission, a M7 robot in the 9th and an M7 and a Raven robot in the 12th mission. Some surgical tasks were explored with the aid of telementoring and telerobotic surgical technology.

In the 7th, partecipants were given minimal surgical training to evaluate the usefulness of telementoring. The 9th NEEMO was focused on real-time abdominal surgery on a patient simulator for developing crew skills. Signal latency, setup to 750 ms, and a delay up to 3 s, were used to mimic the Moon-Earth communication links. In the 12th mission, some sutures were performed on a phantom in simulated zero-gravity conditions by three surgeons guiding the robot’s movements via computer from a remote location. All missions demonstrated the value of telementoring and the feasibility of remote teleoperated surgery, provided that effective communication connections are available^32–35^.

### Studies on robot-assisted minimally invasive surgery (RAMIS)

As reported above, the use of a surgical robot could be of advantage in space surgery. It is well known on Earth that robotic technology is able to extend the surgeon’s dexterity and capabilities to perform many types of complex procedures through tiny incisions with the aid of dedicated surgical instruments. The surgical robots used in clinical activity are master–slave telesurgery devices (Robotic-Assisted Minimally Invasive Surgery-RAMIS) entirely teleoperated by the surgeon, in charge of higher level planning and cognitive decision-making, while robots are responsible only for mechanical implementation^36,37^. Its peculiarity is the presence of “a human-in-the-loop-control”^5,6,36,37^ and safety is provided by the surgeon performing the procedure. An evolution of this condition is the possibility to benefit from special features and effectors^5^. Haidegger^6^, in his paper of 2019, made an excursus on the evolution of surgical robot autonomy. Until today the teleoperated systems in use, although they offer new healing solutions for complex diseases, are not capable of autonomous task execution or cognitive decision-making. The introduction of more difficult procedures and technological advancement in Computer-Integrated Surgery (i.e., “the field of interventional medical technologies, from medical image guidance and augmented reality (AR) applications to automated tissue ablation”)^6^ has led to the development of Human-Robot Interaction, i.e., the transfer of task related knowledge between humans and robots, which represents a major advancement in this field. This effort is strongly related to space surgery, as the presence of a qualified full-trained CMO cannot be taken for granted and even when he/she is present, an assistant to support her/him is mandatory, as stated by Gao et al.^38^. These Authors reported that, in addition to advances in robotics that have allowed the exploration in harsh environments in space, supporting astronauts operations, Robotics has helped to significantly reduce the cognitive load on humans abundance of critical decisions that must be taken in a timely manner to ensure safety^39^. Depending on the distances between the spacecraft and the Ground Control Center, different telepresence technologies may provide the best performance^30,36,37^ among telesurgery, image-guided surgery and cooperatively controlled surgical robotics. Pre-and-intraoperative imaging and physiological data collection shall supply the surgical robot with information to gain more autonomy, future target of Earth and space research^30,36,37^. Takács et al.^36,37^ reported on the signal delay between the Earth and the Moon, and between Earth and Mars. Semi-real-time telesurgery can be used within Earth-Moon distance. In case of a surgical emergency far from the Earth’s orbit, alternative solutions are needed to maintain tele surgery feasibility up to 2 s delay^36,37^. Predictive displays projecting the robot’s intended motions ahead in time up to a maximum of 2 s delay have been considered, stretching human capabilities to the limits, as above 250–300 ms latency surgeons perform worse^36,37^.

The opportunity to have a trained CMO to all spacecraft patients on the spacecraft in deep space mission, is considered the best option in literature. Ground aid shall be given by advisor surgeons, plus storing-and forwarding data^6,35^, exchange of still images, motion videos, voice conferencing, and electronic chats to complete analyses. The target is to have an “intelligent medical system” that will help the CMO with the diagnosis, monitoring and treatment of sick crew members^6,35^. The possibility to consult a dedicated library to find support in case of particularly challenging events is also a good option for the CMO. Surgical navigation and augmented-reality systems shall also be available and a number of force and tactile sensors shall be provided to determine tissue mechanical properties and consistencies for dissection^6,36,37^. Regarding the current advances in RAMIS, Cornejo et at.^40^ report on novel achievements in medical robotics and space surgery. The “Space Biosurgeon” is conceived to provide support in tele-operated advanced laparoscopic surgical procedures applied for General and Gastrointestinal robot-assisted surgery.

This system is composed of a console for the surgeon “SurgiConsole” and a robotic platform “Surgi Platform”. The Biosurgeon conceptual design was developed to achieve a natural alignment of eye, hand and instruments, improve surgical motor dexterity, minimize invasiveness, enhanced surgical ergonomics, feasibility, safety, and reduce risks.

In addition other surgical systems for surgical application in long-distance space missions have been proposed. The “trauma pod”, originally designed for military operations and natural disasters for critical diagnostics and prompt life-saving procedures on the seriously wounded^24^, shall be used when ever timely deployment of proper medical personnel is unavailable and the patient cannot be evacuated quickly to an appropriate medical facility. The platform shall be used for securing the airway, inserting an intravenous or intraosseous line, performing hemostasis, manipulating damaged tissue and positioning monitoring devices^24^.

The RAVEN, (Bio Robotics Lab. University of Washington, Seattle WA) with a weight of only 22 kg operating on the same principle as the DaVinci System, has two articulated tendon driven arms and can be easily assembled even by non engineers supplied with communication links for long distance remote control^36,41^.

Other examples of versatile robots such as Robonaut2^42,43^ were tested for use in medical procedures. Researchers at the NASA Johnson Space Center, in collaboration with General Motors and Oceaneering, designated this highly dexterous, humanoid robot for employment in a variety of medical applications, from telemedicine to medical management either in autonomous or teleoperation mode^43^. Moreover, the Florida Institute for Human and Machine Cognition recently organized the “Blue Sky Meeting”, with the objective of exploring the role of robotics in surgery on space exploration flights^42–44^.

The symposium offered an excursus on the possible use of dexterous human-inspired robots as effective medical-surgical assistants and on advances in space surgery.

## Discussion

It is an established assumption that in long-term missions as well as in missions to another planet, acute medical and surgical care need a large amount of autonomy and also a wide medical and surgical knowledge, due to difficulties in communications with Earth that grow with distance. Any unpredictable injury or medical event that may occur outside the terrestrial orbit is a risk for the individual crew member, the entire crew and the mission itself. The impossibility of a prompt return to Earth, as on ISS, prompted a shift of paradigm in how to face emergency in space missions and missions to other planets. As reported by Robertson et al.^21^ careful planning of the most critical health conditions and their treatment can help not only to high light events with the highest potential to adversely impact on missions as well as the greatest potential for survival, but can envision also NTSs that may be necessary to face these conditions.

The absence of gravity in voyages to another planet is also an investigated field. Although there are studies on there adaptation to 1G at there-entry of a mission, potential consequences are hard to figure out^6,45^ after landing on Mars where a lower gravity than on Earth (0.376 g) is present. Atmospheric pressure is a tiny fraction, averaging 7.5 millibars, of what it is on Earth, over 1000 millibars, another variation that has to be taken into consideration.

In addition, since Mars Missions will last ~3 years, considering voyage and time spent on the planet, surgical emergencies or trauma are more likely to occur^18,20,46^. The possibility to use AR^47–49^ and Virtual reality^49^ could be a helpful possibility for astronauts to take care of themselves in this hostile and extremely remote environment. This tools can implement and maintain CMO and crew members skills in surgery and health care^46^.

Advanced developments toward more autonomous systems capable of assisting the crew and the CMO are under investigation.

In space surgery, moving away from Earth, even a qualified full-trained CMO can find herself/himself left alone in the decision-making process. She/he may not necessarily have all the required knowledge to manage any unexpected health event. In such instances, the use of highly innovative computerized resources may be extremely helpful.

In this sense, the improvement of RAMIS encompasses the recent proposal of a “Space Biosurgeon”^40^, a teloperated Robotic Surgical System which requires that both the surgeon and robot be at the same location.

Aside from Robotic surgery and advances in space settlement and exploration, other issues have been highlighted in this paper, like the Blue Sky Meeting indication of the possibility of using dexterous human-inspired robots for space exploration flights^42,43^ to overcome the medical and surgical challenges and consider innovative future applications (AI, Machine Learning).

Summarizing, the desirable applications of a robotic system in a space flight should be the following: firstly, it should be pre-programmed for basic surgical procedures, such as suturing; secondly, it should have an image-guided autonomous system to be able to employ ultrasound, MRI or computed tomography scans and be ready for real-time decision-making^41^. Other tasks shall include providing a support for anesthesia, vital-signs monitoring, and post-operative care.

Finally, dedicated treatment post trauma recovery programs and equipment should be envisioned for astronauts suffering from substantial injuries^50,51^. Such therapies should be planned for long-term missions and, among the instruments on board, a dedicated device (computer hardware and software) to implement them, should also be present, for there is evidence in the literature of therapies and rehab instruments for upper limbs trauma that have been tested on astronauts in conditions of microgravity^50,51^.

Multi functional surgical robots, capable of performing procedures autonomously, are the new frontier. Further research in this field shall provide new insights on human health in space as well as innovative ideas for clinical applications on Earth.

## Methods

A research on PubMed and Medline, Google Scholar, was performed on “Surgery in Space”, “Robotic Surgery in Space”, “Robot-assisted Surgery in Space”, “Telerobotic Surgical System” “Telementoring” “Communications in Space” “Signal Latency” “Microgravity Effects”. Articles were jointly selected and their references searched by the Authors. The European Space Agency (ESA) and National Aeronautic and Space Administration (NASA) sites were also searched on the following topics: “Deep Space Gate”, “Missions to Mars”, “Space explorations”, “capabilities for exploration spaceflight”, “surgical robot”, “crew health”.
